# Dependence of Wenzel–Cassie Transition on Droplet Size: The Critical Water Droplet

**DOI:** 10.3390/ma19061262

**Published:** 2026-03-23

**Authors:** Mengdan You, Yanfei Wang, Yuzhen Liu, Qiang Sun

**Affiliations:** Key Laboratory of Orogenic Belts and Crustal Evolution, Ministry of Education, The School of Earth and Space Sciences, Peking University, Beijing 100871, China; 2501210162@stu.pku.edu.cn (M.Y.); 2501210166@stu.pku.edu.cn (Y.W.); liuyuzhen@stu.pku.edu.cn (Y.L.)

**Keywords:** Wenzel–Cassie transition, wetting parameter, critical roughness, critical water droplet, hydrogen bonding

## Abstract

In this work, molecular dynamics (MD) simulations are applied to investigate the dependence of the Wenzel–Cassie transition on water droplet size. During the Wenzel–Cassie transition, the critical water droplet and corresponding critical roughness may be expected, which are respectively described as the critical radius (*R*_Droplet,c_) and wetting parameter (*W*_Roughness,c_). From the work, *R*_Droplet,c_ may be termed as the smallest droplet size at which the Cassie state is expected for the corresponding *W*_Roughness,c_. In combination with the structural study of water, it is due to the structural competition between interfacial and bulk water. Additionally, *R*_Droplet,c_ may be dependent on the *W*_Roughness,c_. It is found that the *R*_Droplet,c_ is influenced by the distribution and geometric characteristics of surface roughness. A denser distribution of roughness is expected to result in a lower *R*_Droplet,c_. Consequently, superhydrophobicity may be influenced by the characteristics of surface roughness and the size of the water droplet. The Cassie state is achieved when the wetting parameter of roughness is less than the *W*_Roughness,c_ and the water droplet is larger than the *R*_Droplet,c_.

## 1. Introduction

Wettability, a critical characteristic of solid surfaces, is quantified by the contact angle (CA) formed by a liquid drop. Based on the Young equation [1], the CA is defined as: cos*θ* = (*γ*_SG_ − *γ*_SL_)/*γ*_LG_, where γ_SL_, γ_SG_, and γ_LG_ mean the solid–liquid, solid–gas, and liquid–gas interfacial tensions, respectively. Based on the measured CAs, surfaces are categorized as hydrophilic (<90°) or hydrophobic (>90°) [2]. Recently, due to the multifunctional properties, much attention has been paid to superhydrophobic surfaces, which are defined by a CA greater than 150° and low contact angle hysteresis (CAH < 10°).

The wettability of a solid surface is significantly influenced by its roughness [3], leading to the development of two primary models to describe this effect: the Wenzel model and the Cassie–Baxter (CB) model. In the Wenzel state, the liquid completely penetrates the surface grooves, resulting in enhanced wetting. The apparent contact angle is given by cosθ* = *r*·cos*θ*, in which *r* is the roughness factor, defined as the ratio of the actual solid surface area to the projected smooth surface area [4,5]. In the Cassie state, the droplet rests atop the surface asperities, trapping air pockets beneath it. The CA is expressed as cos*θ*_CB_ = *f*_sl_·cos*θ*_sl_ + *f*_la_·cos*θ*_la_, where *f*_sl_ are the fractional areas of the solid–liquid interface, *f*_la_ means the fractional areas of liquid–air interfaces, and *θ*_sl_ and *θ*_la_ are the respective intrinsic contact angles at these interfaces [6]. Additionally, in our recent works [7,8], a wetting parameter (*W*_Roughness_), related to the ratio of surface area to volume of roughness was proposed to understand the effects of surface roughness on superhydrophobicity. Furthermore, a critical wetting parameter (*W*_Roughness,c_) is found during the Wenzel–Cassie (W–C) wetting transition.

It is found that the Cassie–Wenzel transition may be fulfilled by various methods, which may be divided into external driving [9,10,11,12,13,14,15,16] and self-drop evaporation. In 2005, McHale et al. [17] observed droplet collapse, characteristic of the Cassie–Wenzel transition, during evaporation on periodic micro-column surfaces. Since then, many works [18,19,20,21] have been carried out. This means that evaporating droplets on rough surfaces spontaneously transition from the Cassie state to the Wenzel state once their volume falls below a critical threshold. Additionally, other studies [22,23] have focused on the influence of surface roughness on this critical radius. For instance, Jung et al. [22] investigated how micro-column dimensions (diameter, height, spacing) affected the stability of the Cassie state during evaporation. Consequently, during the Cassie–Wenzel wetting transition, a critical droplet size can be anticipated during the evaporation of a water droplet.

Many studies [24,25,26,27,28,29,30,31] have also investigated the W–C wetting transition. Notably, the W–C wetting transition is found during condensation of the water droplet [32]. In 2009, Boreyko and Chen reported the phenomenon of condensation-induced droplet jumping [33]. During the coalescence of droplets on superhydrophobic surfaces, the release of surface energy provides the driving force for this spontaneous jumping behavior. Consequently, coalescence-induced droplet jumping on superhydrophobic surfaces has garnered considerable attention [34,35,36,37,38,39,40,41,42,43,44,45,46,47,48,49]. In Zhang et al.’s work [48], droplet jumping capacity may be related to droplet radius.

This work aims to investigate the effects of droplet size on the W–C transition. Based on molecular dynamics (MD) simulations, during the W–C transition, the critical droplet size and roughness may be found, which are expressed as *R*_Droplet,c_ and *W*_Roughness,c_. According to the structural analysis, this is ascribed to the competition between bulk and interfacial water. Therefore, superhydrophobicity is related to the characteristics of roughness and the size of the water droplet. The Cassie state may be achieved as the *W*_Roughness_ being less than *W*_Roughness,c_ (<*W*_Roughness,c_), and the size of the water droplet being larger than *R*_Droplet,c_ (>*R*_Droplet,c_).

## 2. Methods

### 2.1. MD Simulations

To investigate the dependence of the W–C transition on water droplet size, MD simulations were conducted through the GROMACS package (version: Gromacs 2019.6) [50,51]. During the simulations, the OPLS–AA force field was utilized to model the interactions between carbon atoms in the graphite substrate. Due to its high accuracy in capturing both the thermodynamic properties of water [52,53] and the properties of water at surfaces and interfaces [54], the TIP4P/2005 model [55] was utilized for water simulation.

The TIP4P/2005 model [55] describes intermolecular interactions through the Lennard–Jones (LJ) term, *U*_LJ_, and the electrostatic term, *U*_elec_. The LJ potential may be expressed as

(1) $$U_{LJ\left(r_{ij}\right)}=4\varepsilon_{ij}\left[{\left(\frac{\sigma_{ij}}{r_{ij}}\right)}^{12}-{\left(\frac{\sigma_{ij}}{r_{ij}}\right)}^{6}\right]$$

where *ε* and *σ* mean the energy and length parameters, and *r*_ij_ is the distance between molecules *i* and *j*. The electrostatic term is described by Coulomb’s law as

(2) $$U_{elec}=\frac{e^{2}}{4\pi \varepsilon_{0}}\sum_{ij}\frac{q_{i}q_{j}}{r_{ij}}$$

where *ε*_0_ is the vacuum permittivity, *e* means the elementary charge, and *q*_i_ and *q*_j_ represent the partial charges on atoms *i* and *j*. The parameters of the TIP4P/2005 water model are listed in Table 1.

Simulations were performed in a cubic box of approximately 16.0 nm × 16.0 nm × 16.0 nm. During the simulations, the graphite substrate was fixed. The canonical (NVT) ensemble was used. The temperature was kept at 300 K using a Nosé–Hoover thermostat. Periodic boundary conditions were imposed in all directions. Interactions were modeled using Lennard–Jones potentials truncated at a cutoff distance of 1.0 nm, while long-range electrostatic interactions were calculated precisely using the Particle Mesh Ewald (PME) method. During the simulations, once the potential energy (or total energy) reaches a constant value, equilibrium has been reached. For each simulation, the simulated time was 6 ns using 2 fs time step.

**Figure 1:**
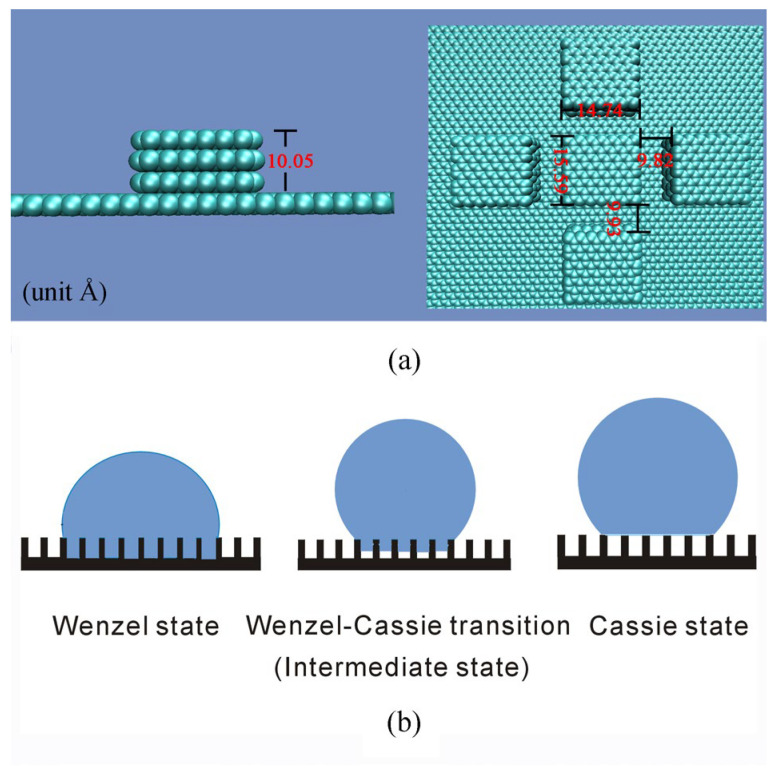
(**a**) Characteristics of surface roughness I–H3. (**b**) During the W–C transition, this decreases the roughness–water interface.

In the simulations, square pillars are used to study the roughness effects on superhydrophobicity. The pillar side lengths in the x and y axes are denoted as *a*_x_, *a*_y_, the groove widths between pillars in the x and y axes are expressed as *w*_x_, *w*_y_, and the pillar height is *h* (Figure 1 and Table 2).

### 2.2. CA Measurement

An ImageJ package (https://imagej.net/ij/download.html (accessed on 6 July 2023)) [56] with a contact angle plugin was applied to determine the CAs. After achieving thermodynamic equilibrium, a near-spherical water droplet (radius about 4.0 nm) was found on the substrate. To determine the CA, a manual selection procedure was employed: the baseline was defined by two points, and the droplet profile was identified by three points. These points were used to determine the droplet’s contour and used for CA measurement. For each droplet, CAs were measured three times. The uncertainty of CAs was about 3°.

### 2.3. Hydrogen Bondings and Density

As a water droplet rests on a rough surface, an interface is formed. In our recent works [7,8], this roughness–water interface significantly affects the hydrogen bonds of the topmost water molecular layer, defined as interfacial water. This is also demonstrated by structural studies [57,58,59,60] concerning the solvation of ions, which explain that ionic effects are predominantly localized within the first hydration shell. To understand the roughness effects on water structure, both hydrogen bonding and density distributions were also calculated using the Visual Molecular Dynamics (VMD) program (version: 1.9.3) [61].

Water is generally regarded as an anomalous liquid, which is related to the formation of hydrogen bonds (HBs) between water molecules. To study the changes of hydrogen bonding during the W–C transition, a geometrical definition is applied to determine the number of HBs in liquid water [62]. Hydrogen bonding is considered to exist between two water molecules if the distance between the two oxygen atoms (*r*_OO_) is smaller than 3.5 Å and the ∠OOH angle is less than 30°.

## 3. Results

**Figure 2:**
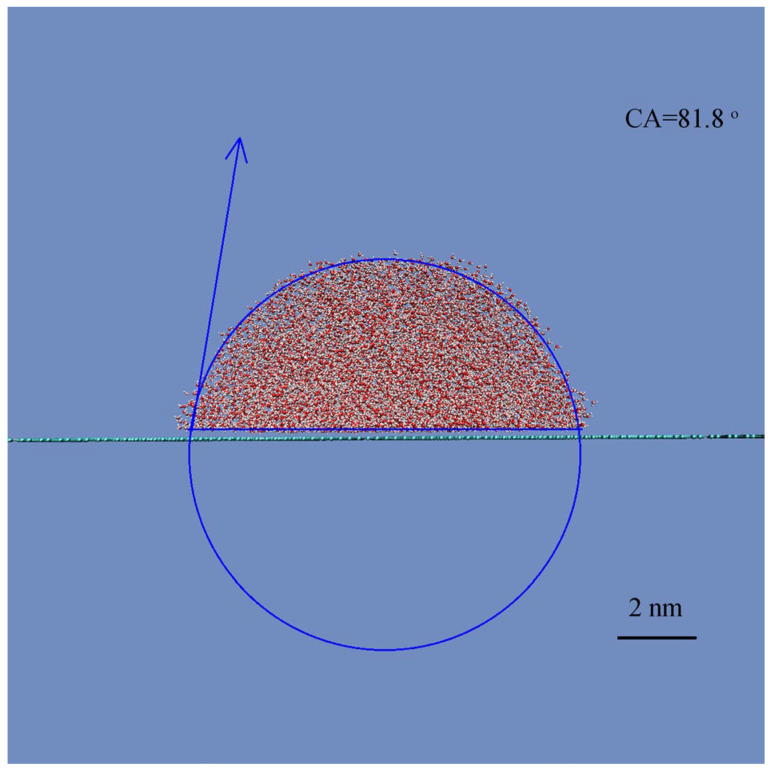
CA measurement on a smooth graphite surface.

This study aims to investigate the effects of water droplet size on the W–C transition. In the study, we first conducted MD simulations to determine the static CA on a smooth graphene surface. The CA is measured to be 81.8° (Figure 2), which is consistent with the typically reported range of 75° to 95° for graphitic surfaces [63]. This may be used as the reference to study the roughness effects on superhydrophobicity.

**Figure 3:**
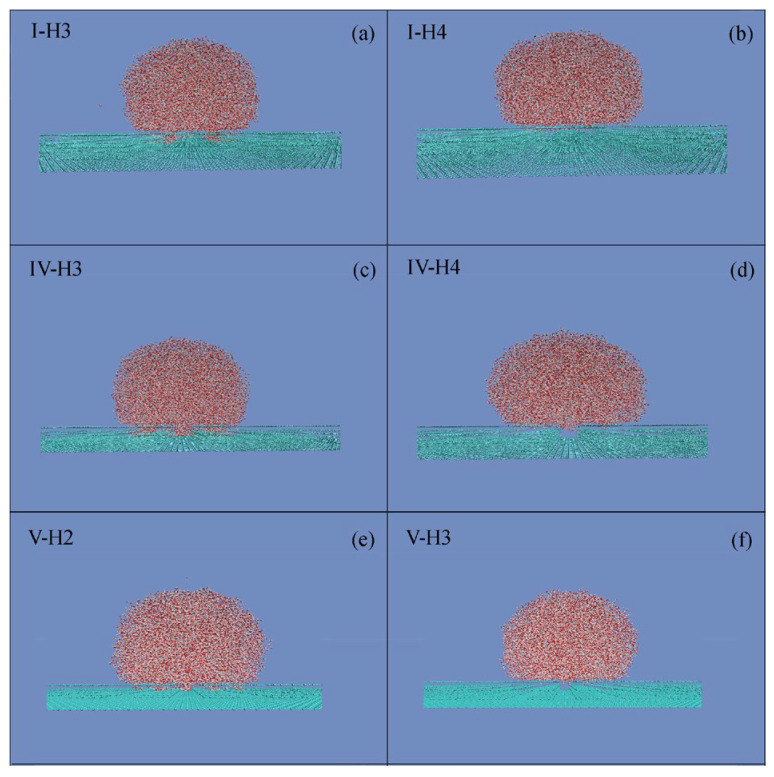
(**a**–**f**) During the simulations, a 7.0 × 7.0 × 7.0 nm^3^ cubic water box was placed on various surfaces to study the roughness effects on the W–C transition. The characteristics of surface roughness are listed in Table 2.

To investigate the roughness effects on superhydropobicity, the square roughnesses are added on the solid surface. With reference to smooth graphite, CAs may be influenced by the square roughness (Table 2). With increasing the roughness height (*h*), this increases the measured CAs. Additionally, during the W–C wetting transition, a sharp increase may be found for CA (Figure 3 and Table 2).

During the MD simulations, a cubic water box (7.0 × 7.0 × 7.0 nm^3^) was initially placed above a graphite substrate patterned with various roughness configurations (Table 2). As equilibrium was reached, the liquid was engaged in a Wenzel or Cassie state. In our recent works [7,8], *W*_Roughness_, the ratio of surface area to volume of surface roughness, was proposed to understand the roughness effects on superhydrophobicity. During the W–C wetting transition, a critical surface roughness, *W*_Roughness,c_, could be determined (Figure 3 and Table 2). Consequently, the Cassie state is expected for *W*_Roughness_ < *W*_Roughness,c_, while the Wenzel state is observed for *W*_Roughness_ > *W*_Roughness,c_.

**Figure 4:**
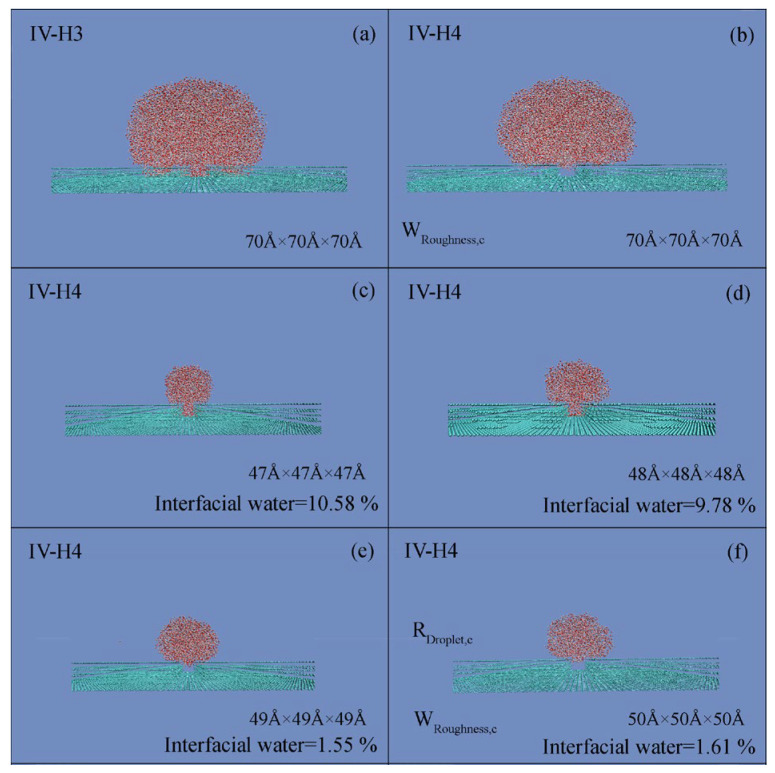
Critical water droplet. (**a**,**b**) During the simulations, a 7.0 × 7.0 × 7.0 nm^3^ cubic water droplet was employed to determine the *W*_Roughness,c_ for each setup. (**c**–**f**) Regarding the *W*_Roughness,c_, as the size of the initial cubic water box was incrementally increased (by 1 Å steps), the W–C transition was observed for the *W*_Roughness,c_. This may be applied to determine the *R*_Droplet,c_ related to the *W*_Roughness,c_.

During the simulations, to study the dependence of the Cassie state on the droplet size, various cubic water boxes were respectively placed on this critical roughness (*W*_Roughness,c_) (Figure 4). With increasing the droplet size, the W–C wetting transition is found. Consequently, the superhydrophobicity is influenced by the size of the water droplet.

Regarding a given *W*_Roughness,c_, the Wenzel state is found when the droplet size is less than a threshold value of a water droplet, and the Cassie state may be expected when the droplet size is larger than the threshold. Consequently, during the W–C wetting transition, a critical size of a water droplet may be expected for the given *W*_Roughness,c_. In the work, this is termed as the critical water droplet, which means the smallest droplet size at which the Cassie state can be exhibited on the critical *W*_Roughness,c_. Additionally, during the MD simulations, because the radius of the water droplet is easily measured, the measured critical droplet radius is used to represent the size of a critical water droplet, *R*_Droplet,c_ (Table 3).

**Figure 5:**
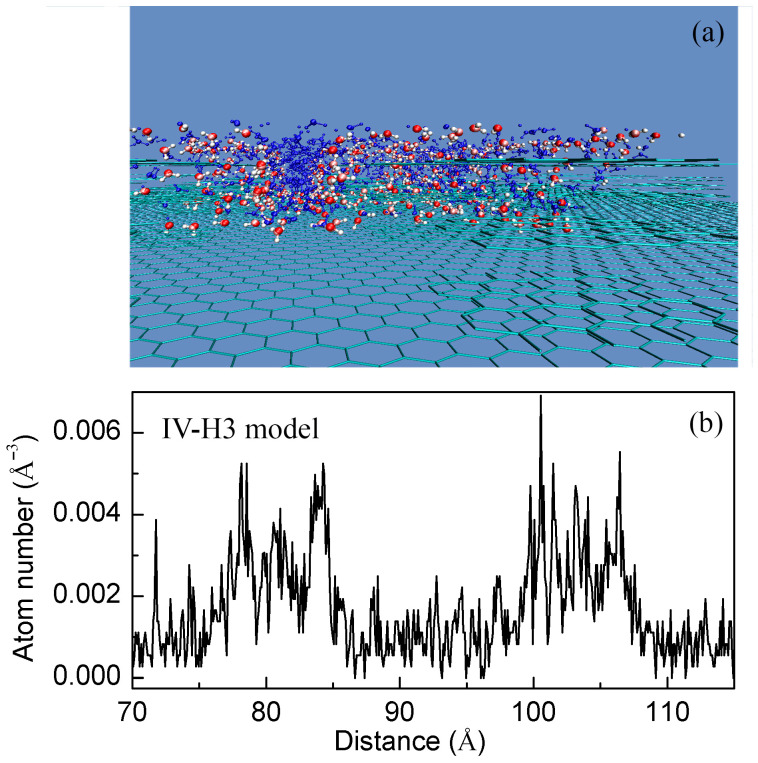
Density distribution of water molecules. (**a**) Roughness mainly affects the structure of interfacial water. Interfacial and bulk water molecules are respectively shown in CPK representation and blue. (**b**) Compared to the bulk water, a higher density is found for the interfacial water.

**Figure 6:**
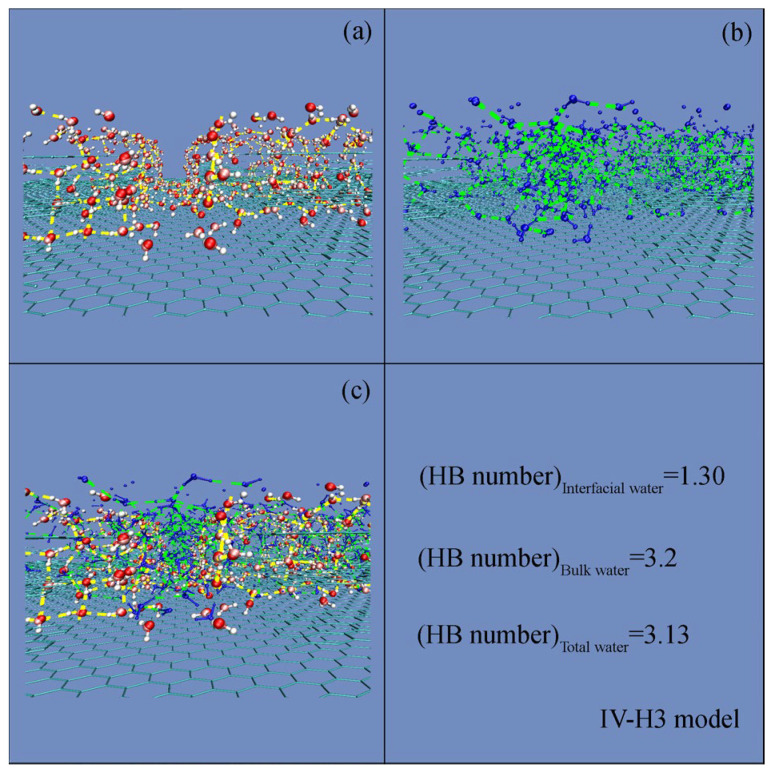
Hydrogen bondings for interfacial (**a**), bulk (**b**) and total water (**c**). Hydrogen bonds of water are shown in dashed lines.

In the Wenzel state, liquid water fully occupies the surface indentations and the roughness is embedded within the liquid. Conversely, in the Cassie state, a water droplet does not penetrate the surface grooves, and air is trapped within them, forming a heterogeneous interface. During the W–C wetting transition, it is characterized by a decrease of the roughness–water contact area. To elucidate the mechanism of the W–C transition, it is crucial to study the roughness effects on the water structure. Based on the MD simulations, the density distribution (Figure 5) and hydrogen bonding (Figure 6) were respectively calculated.

From Figure 5, compared to bulk water, a higher density may be found for water molecules at the roughness–water interface. It is also consistent with other experimental measurements [64] and theoretical simulations [65] on confined water. This means that the roughness mainly affects the structure of the interfacial water layer. Additionally, obvious structural difference may be observed between the bulk and interfacial water.

Additionally, based on the geometric definition of hydrogen bonding [62], the hydrogen bonding number was respectively calculated for interfacial, bulk, and total water (Figure 6). Our results indicate that bulk water exhibits a higher average number of hydrogen bonds than interfacial water. This reduction is attributed to the truncation of the hydrogen bonding network at the roughness–water interface. Moreover, compared to bulk water, hydrogen bonding in the interfacial layer is dominated by single donor–single acceptor (DA) configurations [7,8].

**Figure 7:**
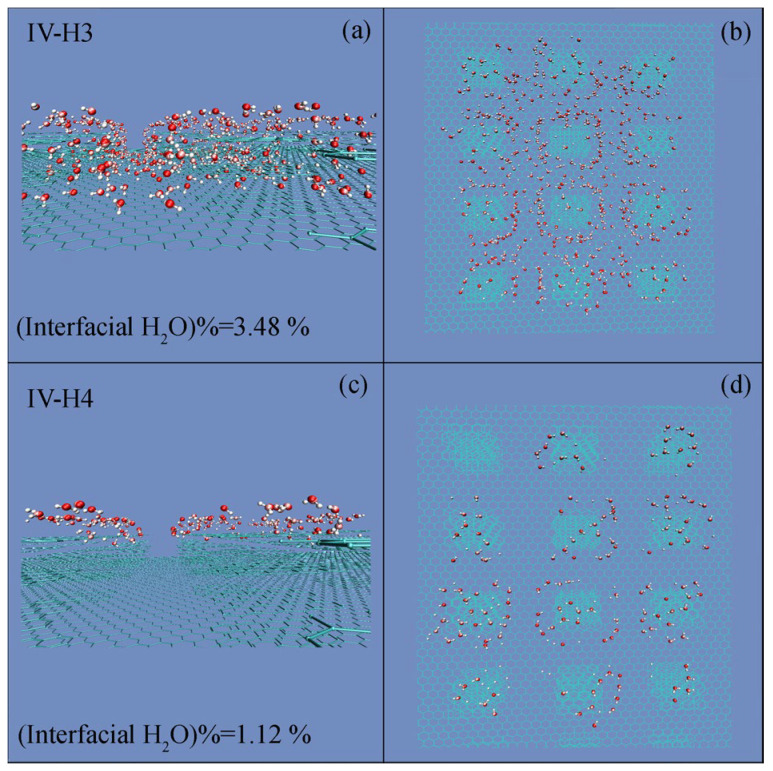
(**a**–**d**) The changes in interfacial water during W–C transition. Side and top views are presented.

The roughness primarily affects the hydrogen bonds of interfacial water. During the simulations, interfacial water is defined as water molecules within 3.8 Å of the surface of graphite. During the W–C wetting transition, this decreases interfacial water (Figure 7) and increases bulk water. These indicate that the W–C transition is associated with the structural reorganization between bulk and interfacial water.

From the above, supherhydrophobicity may be influenced by surface roughness (Table 2); the Cassie state is fulfilled when the *W*_Roughness_ is smaller than *W*_Roughness,c_. Additionally, with increasing the droplet size, the *R*_Droplet,c_ was found, in which the W–C wetting transition was observed (Table 3). Therefore, the W–C wetting transition may be influenced by both the geometric characteristics of roughness and the droplet size.

**Figure 8:**
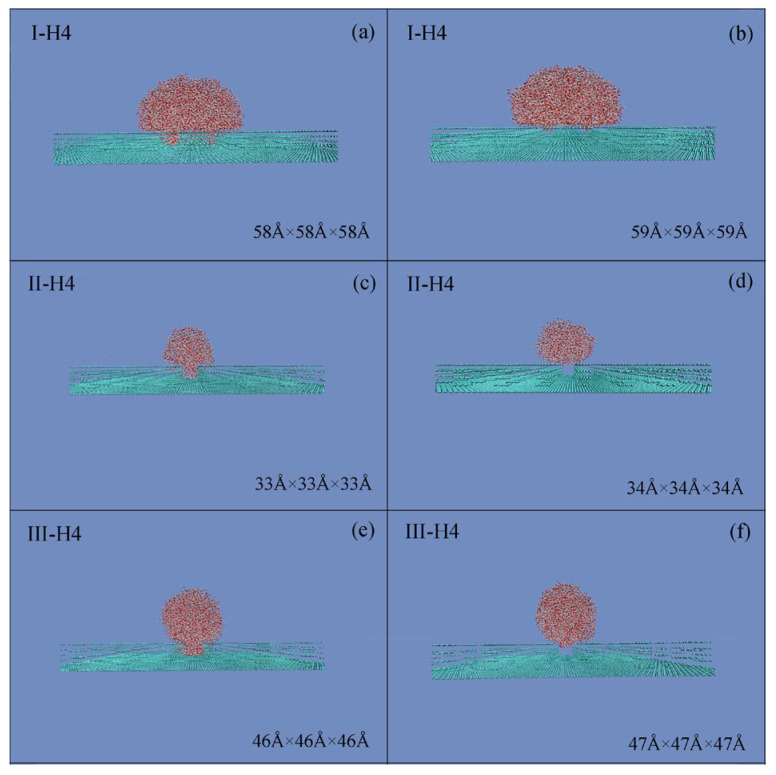
(**a**–**f**) The dependence of *R*_Droplet,c_ on *W*_Roughness,c_. Various critical water droplets may be found for different critical roughness.

In the work, various square-surface roughness configurations are applied to investigate the effects of droplet size on the W–C transition (Table 3). From the work, the corresponding relationship between *W*_Roughness,c_ and *R*_Droplet,c_ may be found (Figure 8). In other words, this means that various *R*_Droplet,c_ may be expected for different *W*_Roughness,c_ (Table 3).

**Figure 9:**
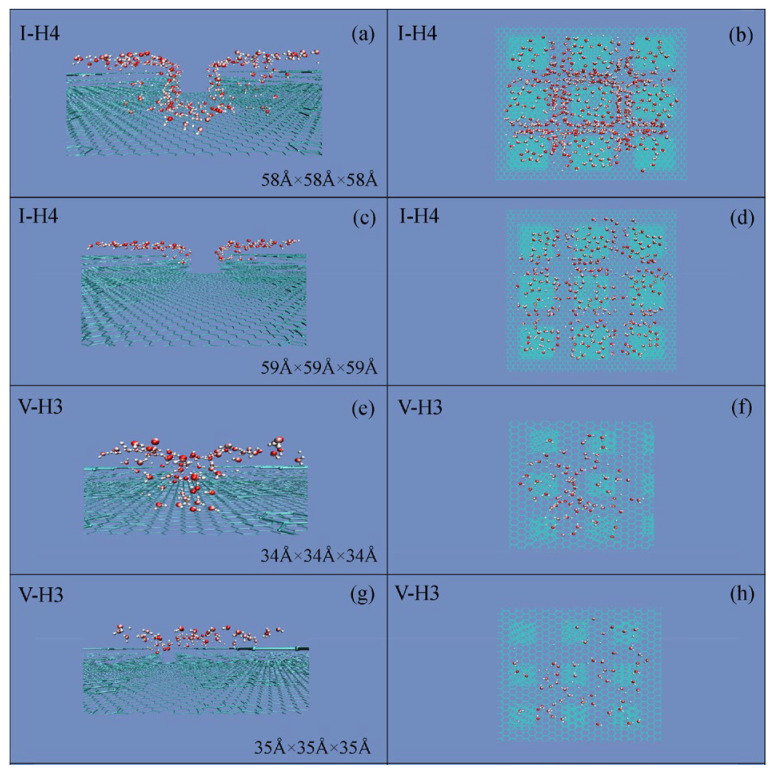
(**a**–**h**) The changes in interfacial water during the W–C transition. Interfacial water molecules are shown in CPK representation. Side and top views are presented.

Because roughness significantly affects the hydrogen bonds of interfacial water, *R*_Droplet,c_ may be dependent on the geometric properties of the critical roughness. From the work, the changes in interfacial water may be determined for various critical water droplets (Figure 9). It is found that a sharp decrease may be observed for interfacial water when a water droplet approaches the *R*_Droplet,c_ (Figure 9). This means that the *R*_Droplet,c_ is dependent on the distribution of interfacial water on the surface roughness. For instance, a denser distribution of surface roughness may lead to a smaller critical droplet size.

During the W–C wetting transition, the critical water droplet and corresponding critical roughness may be expected. Based on the MD simulations, the *R*_Droplet,c_ is dependent on interfacial water, which is affected by the distribution and geometric features of surface roughness. Consequently, superhydrophobicity is influenced by the surface roughness, and the size of the water droplet. Additionally, the Cassie state is found as the wetting parameter of roughness being less than the *W*_Roughness,c_ and the water droplet size being larger than *R*_Droplet,c_.

## 4. Discussion

During the W–C wetting transition, for a given *W*_Roughness,c_, the Cassie state emerges when the water droplet size exceeds a critical threshold (Figure 4). From the work, the *R*_Droplet,c_ may be found, which is defined as the smallest water droplet at which the Cassie state can be exhibited on the critical roughness. Consequently, the W–C transition is related to the characteristics of surface roughness, and the size of the water droplet.

During the W–C wetting transition, this is accompanied with the decrease of the roughness–water contact area (Figure 9). Because surface roughness mainly affects the hydrogen bonds of interfacial water, the W–C transition may be closely related to the structural rearrangement of water molecules. This may provide an approach to understand the physical origin of the critical water droplet.

Extensive research has been conducted to elucidate the structure of liquid water. To date, various theoretical models have been proposed to describe this structure, which can be broadly categorized into mixture models and continuum models [66,67]. In our Raman spectroscopic studies [68,69], various local hydrogen-bonded arrangements may be found around a water molecule, such as DDAA (double donors–double acceptors, tetrahedral hydrogen bonding), DDA, DAA, and DA configurations [69].

When a water droplet contacts surface roughness, this is divided into bulk and interfacial water. In comparison with bulk water, higher density and fewer hydrogen bonds are expected for interfacial water (Figure 5 and Figure 6). Obvious structural differences may be found between them. Additionally, the formation of a roughness–water interface is attributed to the loss of the DDAA (tetrahedral) structural motif in interfacial water [7,70]. Therefore, Gibbs free energy of interfacial water (Δ*G*_Roughness–water_) can be given as,

(3) $$\begin{array}{l} \Delta G_{\mathrm{Roughness}-\mathrm{water}}=R_{\mathrm{Interfacialwater}/\mathrm{bulkwater}}\cdot \Delta G_{\mathrm{DDAA}}\cdot n_{\mathrm{DDAA}}\\ \;\;=\frac{\left(\mathrm{Surface}\;\mathrm{area}\right)/\left(\pi r_{H2O}^{2}\right)}{\left(\mathrm{Volume}\right)/\left(\frac{4}{3}\pi r_{H2O}^{3}\right)}\cdot \Delta G_{\mathrm{DDAA}}\cdot n_{\mathrm{DDAA}} \end{array}$$

where Δ*G*_DDAA_ represents Gibbs free energy of DDAA hydrogen bonds, *R*_Interfacial water/Bulk water_ means the ratio of interfacial to bulk water for a roughness, *n*_DDAA_ is the average hydrogen bonding number per water molecule, and *r*_H2O_ means the radius of a water molecule.

During the W–C wetting transition, it is related to the decrease in interfacial water, and accompanied with the increase in bulk water. In thermodynamics, this structural rearrangement between them may be described by,

(4) $$\Delta G_{\mathrm{Roughness}-\mathrm{water}}=\Delta G_{\mathrm{Water}-\mathrm{water}}\;\;\;\;(\mathrm{Critical}\;\mathrm{water}\;\mathrm{droplet})$$

When Δ*G*_Roughness–water_ is less than Δ*G*_Water–water_, the Wenzel state is expected, with a preference for interfacial water. Conversely, the Cassie state, which minimizes interfacial water, is expected when Δ*G*_Roughness–water_ > Δ*G*_Water–water_. This may be used to understand the origin of the critical water droplet.

Based on Equations (3) and (4), the following relationship can be derived for the wetting transition from Wenzel to Cassie states

(5) $$\underset{W_{\mathrm{Roughness}}}{\underset{︸}{{\left(\frac{\mathrm{Surfacearea}}{\mathrm{Volume}}\right)}_{\mathrm{Roughness}}}}=\underset{W_{\mathrm{Water}}}{\underset{︸}{\frac{3}{8r_{H2O}}\cdot \frac{\Delta G_{\mathrm{Water}-\mathrm{water}}}{\Delta G_{\mathrm{DDAA}}}}}$$

From the equation, *W*_Roughness_ and *W*_Water_ may be proposed, which are respectively defined as the wetting parameters of roughness and water.

During the W–C transition, the critical wetting parameters are expected and denoted as *W*_Roughness,c_ and *W*_Water,c_, respectively. From these, *R*_Droplet,c_ is expected during the W–C transition. Additionally, the critical water droplet can be defined as the smallest droplet for which the Cassie state emerges at *W*_Roughness,c_ (Figure 4). Regarding the origin of the critical droplet, it is due to the competition between bulk and interfacial water. Based on our recent works [71,72], hydrophobic interactions may play an important role during the W–C transition.

From Equation (5), it is also derived that there exists a corresponding relationship between *W*_Roughness,c_ and *W*_Water,c_. In other words, various critical water droplets may be expected for the corresponding critical roughness. For instance, based on the MD simulations, *R*_Droplet,c_ may be determined to be 21.2 Å and 30.0 Å for II–H4 and IV–H4 roughnesses, respectively (Figure 8 and Table 3).

**Figure 10:**
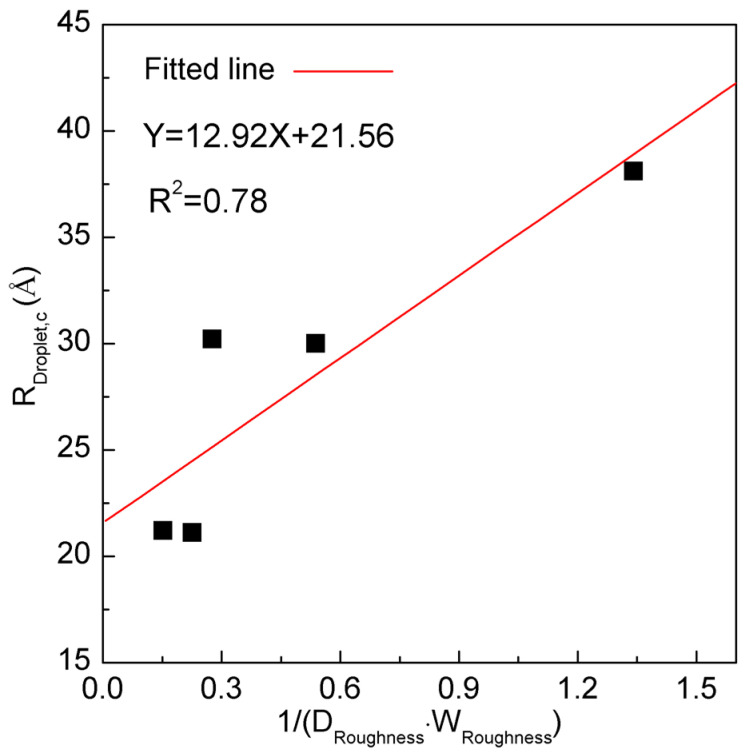
The dependence of critical water droplet on the distribution and *W*_Roughness_ of surface roughness.

From the above, the W–C transition, related to *W*_Roughness,c_ and *W*_Water,c_, may be ascribed to the reorganization between bulk and interfacial water (Figure 9). Consequently, it is derived that the critical water droplet is influenced by the roughness distribution, and the characteristics of roughness (Figure 10).

In the work, roughness distribution means the number of roughnesses per unit surface area. Regarding the square roughness, it may be defined as *D*_Roughness_ = 1/((*a*_x_ + *w*_x_)·(*a*_y_ + *w*_y_)) (Table 3). For the effects of roughness on interfacial water, this is related to the side lengths, separations and roughness height, which may be measured by the wetting parameter of roughness, *W*_Roughness_. Consequently, the following relationship may be derived

(6) $$R_{\mathrm{Droplet},c}\propto \frac{1}{D_{\mathrm{Roughness}}}\cdot \frac{1}{W_{\mathrm{Roughness}}}$$

From Figure 10, with increasing *D*_Roughness_ and *W*_Roughness_, the interfacial water related to roughness increases, and the size of the critical water droplet decreases. For instance, a denser distribution of surface roughness was found to correlate with a smaller critical droplet size (Figure 10 and Table 3).

From the thermodynamic analysis on the W–C wetting transition, it is characterized by *W*_Roughness,c_ and *W*_Water,c_. Consequently, superhydrophobicity is influenced by the geometric characteristics of roughness and the size of the water droplet. With reference to *W*_Roughness,c_ and *R*_Droplet,c_, it may be expressed as

(7) $$\begin{array}{l} \mathrm{Wenzel}\;\mathrm{state}:W_{\mathrm{Roughness}}>W_{\mathrm{Roughness},c}\;\mathrm{and}\;\mathrm{Droplet}<R_{\mathrm{Droplet},c}\\ \;W_{\mathrm{Roughness}}=W_{\mathrm{Roughness},c}\;\mathrm{and}\;\mathrm{Droplet}<R_{\mathrm{Droplet},c}\\ \;W_{\mathrm{Roughness}}>W_{\mathrm{Roughness},c}\;\mathrm{and}\;\mathrm{Droplet}=R_{\mathrm{Droplet},c}\\ \mathrm{Wenzel}-\;\mathrm{Cassie}\mathrm{transition}:W_{\mathrm{Roughness}}=W_{\mathrm{Roughness},c}\;\mathrm{and}\;\mathrm{Droplet}=R_{\mathrm{Droplet},c\;}\\ \mathrm{Cassie}\;\mathrm{state}:W_{\mathrm{Roughness}}<W_{\mathrm{Roughness},c}\;\mathrm{and}\;\mathrm{Droplet}>R_{\mathrm{Droplet},c}\\ \;W_{\mathrm{Roughness}}=W_{\mathrm{Roughness},c}\;\mathrm{and}\;\mathrm{Droplet}>R_{\mathrm{Droplet},c}\\ \;W_{\mathrm{Roughness}}<W_{\mathrm{Roughness},c}\;\mathrm{and}\;\mathrm{Droplet}=R_{\mathrm{Droplet},c} \end{array}$$

Based on Equation (7), the Cassie state is anticipated when the surface roughness falls below a critical value (*W*_Roughness_ < *W*_Roughness,c_) and the droplet size exceeds a critical value (*R*_Droplet_ > *R*_Droplet,c_).

**Figure 11:**
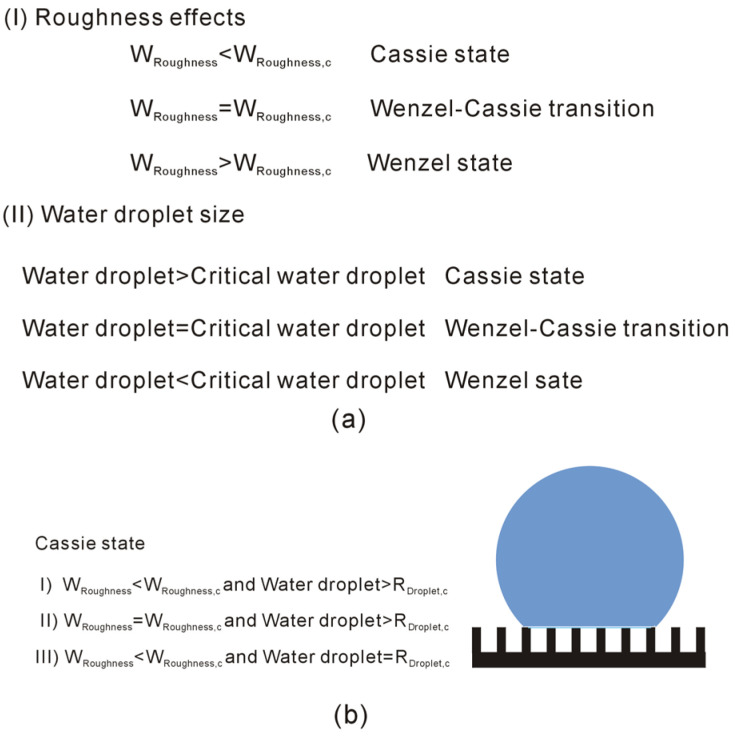
The dependence of superhydrophobicity on *W*_Roughness,c_ and *R*_Droplet,c_. (**a**) Superhydrophobicity may be influenced by surface roughness and water droplet. (**b**) Cassie state is expected when *W*_Roughness_ is less than *W*_Roughness,c_, and water droplet is larger than *R*_Droplet,c_.

From the work, the W–C transition is dependent on the *W*_Roughness,c_ and *R*_Droplet,c_ (Figure 11). Additionally, there exists a corresponding relationship between them. Consequently, modulating the W–C transition requires accounting for the interdependence of these two geometric parameters: surface roughness characteristics and droplet size.

## 5. Conclusions

This work aims to investigate the dependence of the W–C transition on water droplet size. From this work, the following conclusions are derived:

(1) During the W–C wetting transition, the *R*_Droplet,c_ and corresponding *W*_Roughness,c_ may be expected. From the study, *R*_Droplet,c_ can be understood as the smallest droplet size at which the Cassie state is expected for the *W*_Roughness,c_.
(2) Regarding the origin of the critical water droplet, it is due to the structural competition between bulk and interfacial water. In addition, it is found that the *R*_Droplet,c_ is dependent on the *W*_Roughness,c_.
(3) The W–C transition may be affected by the characteristics of surface roughness and the size of a water droplet. The Cassie state is defined as *W*_Roughness_ being less than *W*_Roughness,c_, and the water droplet being larger than the *R*_Droplet,c_.

## Figures and Tables

**Table 1:** Force-field parameters of the TIP4P/2005 water model [55].

Model	∠HOH (°)	*r*_OH_ (Å)	*r*_OM_ (Å)	*q*_H_ (e)	*q*_M_ (e)	*σ*_OO_ (Å)	*ε*_OO_ (kJ·mol^–1^)
TIP4P/2005	104.52	0.9572	0.1546	0.5564	−2 q_H_	3.1589	0.7749

**Table 2:** The geometric characteristics of square roughness. In the study, a cubic water box (7.0 × 7.0 × 7.0 nm^3^) was placed on a graphite substrate to investigate the wettability of the solid surface. The CAs and *W*_Roughness_ are shown during the W–C transition.

System	Model	*a*_x_ (Å)	*a*_y_ (Å)	*w*_x_ (Å)	*w*_y_ (Å)	*h* (Å)	Wettability	CA (°)	*W*_Roughness_
I	I–H4	14.74	15.59	9.82	9.93	13.45	Cassie	131.7	0.46
	I–H3	14.74	15.59	9.82	9.93	10.05	Wenzel	127.3	0.53
II	II–H4	4.91	4.25	12.28	12.76	13.45	Cassie	145.6	1.92
	II–H3	4.91	4.25	12.28	12.76	10.05	Wenzel	143.9	2.26
III	III–H4	4.91	7.09	17.19	14.18	13.45	Cassie	145.8	1.69
	III–H3	4.91	7.09	17.19	14.18	10.05	Wenzel	141.1	2.03
IV	IV–H4	9.82	8.51	12.28	12.05	13.45	Cassie	126.6	0.84
	IV–H3	9.82	8.51	12.28	12.05	10.05	Wenzel	123.7	0.98
V	V–H3	7.37	7.09	7.37	7.80	10.05	Cassie	132.5	0.97
	V–H2	7.37	7.09	7.37	7.80	6.70	Wenzel	128.6	1.18

**Table 3:** The *R*_Droplet,c_ and corresponding *W*_Roughness,c_.

Model	ax (Å)	ay (Å)	wx (Å)	wy (Å)	h (Å)	Wettability	*W*_Roughness,c_	Cubic Water Box (Å^3^)	Distribution (Å^–2^)	*R*_Droplet,c_ (Å)
I–H4	14.74	15.59	9.82	9.93	13.45	Cassie	0.46	59 × 59 × 59	0.001595	38.1
II–H4	4.91	4.25	12.28	12.76	13.45	Cassie	1.92	34 × 34 × 34	0.003418	21.2
III–H4	4.91	7.09	17.19	14.18	13.45	Cassie	1.69	47 × 47 × 47	0.002127	30.2
IV–H4	9.82	8.51	12.28	12.05	13.45	Cassie	0.84	49 × 49 × 49	0.002201	30.0
V–H3	7.37	7.09	7.37	7.80	10.05	Cassie	0.97	35 × 35 × 35	0.004557	21.1

## Data Availability

The original contributions presented in this study are included in the article. Further inquiries can be directed to the corresponding author.

## Abbreviations

The following abbreviations are used in this manuscript:

*W*_Roughness,c_	Critical wetting parameter of roughness
*W*_Water,c_	Critical wetting parameter of water
*R*_Droplet,c_	Critical water droplet radius
*D*_Roughness_	Roughness distribution
